# Anti-tumor effect and immune-related mechanism study of compound aluminum sulfate injection in transplanted tumor-bearing mice

**DOI:** 10.3389/fimmu.2025.1583275

**Published:** 2025-05-01

**Authors:** Zhenwei Shi, Zhifa Xia, Songtao Huang, Zeteng Chen, Fan Yin, Haili Xin, Fenghua Xu

**Affiliations:** ^1^ Medical School of Chinese People’s Liberation Army General Hospital, Beijing, China; ^2^ Pharmaceutical Sciences Research Division, Department of Pharmacy, Medical Supplies Center, Chinese People's Liberation Army General Hospital, Beijing, China; ^3^ The Second Medical Center, Chinese People's Liberation Army General Hospital, Beijing, China; ^4^ Department of Pharmacy, Medical Supplies Center, Chinese People's Liberation Army General Hospital, Beijing, China

**Keywords:** compound aluminum sulfate injection, anti-tumor effect, melanoma, immunomodulator, metastasis

## Abstract

This study investigates the antitumor and immunomodulatory effects of compound aluminum sulfate (CAS) solution in murine melanoma models. Using syngeneic B16-F10 and B16-OVA tumor models, we demonstrate that intratumoral CAS injection significantly inhibits primary tumor growth and lung metastasis. Flow cytometry analysis reveals that CAS treatment increases splenic populations of CD3^+^CD8^+^ cytotoxic T cells, CD3^+^CD44^+^ memory T cells, and NK cells, while enhancing CD8^+^ T cell infiltration in tumor tissue. ELISA results show elevated levels of pro-inflammatory cytokines (IFN-γ, TNF-α, and IL-2) in splenic culture supernatants and serum following CAS administration. Immunofluorescence staining confirms increased expression of CD8 and IFN-γ proteins in tumor tissues of CAS-treated mice. Results indicate that CAS exerts its antitumor effects through direct cytotoxicity and by modulating both systemic and local immune responses. The dual action of CAS, which combines tumor necrosis with immunostimulation, positions it as a promising therapeutic agent for cancer treatment. This study offers valuable insights into the mechanisms underlying CAS’s action and underscores its potential clinical applications in oncology.

## 1 Introduction

Compound aluminum sulfate injection is a local injection drug under phrase III clinical trial in China (CTR20171604, https://www.clinicaltrials.cn). It is primarily used to treat transitional cell tumors of the bladder, particularly non-muscle invasive bladder cancer (NMIBC) (Ta/T1 stage) and certain benign bladder tumors. The drug works by inducing tumor necrosis, which helps to effectively control tumor growth (1). The efficacy and safety of the drug have been verified by *in vitro* experiment and animal test (1). Compound aluminum sulfate injection has significant inhibitory effect on the growth of bladder tumor (1).

Emerging evidence indicates that the therapeutic benefits of intratumoral agents may extend beyond localized cytotoxicity. Many ablative therapies, such as electrocoagulation and cryotherapy, are recognized for their ability to modulate the tumor microenvironment (TME) by releasing tumor-associated antigens (TAAs) and damage-associated molecular patterns (DAMPs). This release can trigger systemic antitumor immune responses (2–6). For instance, aluminum-based compounds have historically been utilized as vaccine adjuvants due to their ability to enhance antigen presentation and stimulate Th1-type immune responses (7–12). Recent advances demonstrate that nanostructured or functionalized aluminum formulations can significantly enhance these immunomodulatory effects. For instance, aluminum oxide nanoparticles have been shown to boost cytotoxic lymphocyte activity and work synergistically with tumor cell vaccines to suppress tumor growth (13–16). Similarly, -1,3-glucan-functionalized aluminum hydroxide nanovaccines activated dendritic cells via Dectin-1 signaling, driving robust CD8^+^ T cell responses and prolonged survival in murine tumor models (11, 17–20). These studies emphasize the potential of engineered aluminum compounds to address the limitations of traditional adjuvants, particularly in enhancing the activation of antigen-presenting cells (APCs) and bridging innate and adaptive antitumor immunity. Furthermore, these findings suggest that CAS, through its aluminum component, may similarly enhance antitumor immunity by modulating immune cell dynamics and cytokine profiles within the TME.

In this study, we employed syngeneic murine melanoma models to systematically evaluate the effects of CAS on tumor growth, metastasis, and immune modulation.

## 2 Materials and methods

### 2.1 Cell culture

B16-F10 and B16-OVA mouse melanoma cells were obtained from the Academy of Military Medical Sciences China and were cultured in 25 cm^2^ and 75cm^2^ culture bottles, 37°C, 5%CO_2_ saturated humidity incubator, and the medium was RPMI 1640 medium containing 10% fetal bovine serum.

### 2.2 Syngeneic tumor and metastasis models

Healthy female C57BL/6 mice aged 6–8 weeks, weighing approximately 20.23 ± 1.72g, purchased from SBF (Beijing) Biotechnology Co., Ltd. and raised in the People’s Liberation Army of China Hospital 301 Hospital Laboratory Animal Center, in the dark cycle of 12-h light/12-h, animals are kept in a standard SPF condition (25°C and 40%-60% relative humidity) environment, and standard food is given normally. The water, squirrel cage, cage cover, lid, feed, and drinking water were autoclaved and disinfected and replaced twice a week. In the initial setup, we included 8 animals per group. Following successful tumor modeling, 5 animals were incorporated into each group for further analysis. Additionally, 5 samples per group were used for flow cytometry analysis. All tests were reviewed and conducted under the supervision of the institutional animal experiment welfare ethics committee (approval no. 2019-X15-90).

Compound aluminum sulfate (CAS) was provided by PLA General Hospital. The collected mouse melanoma cell suspension (B16-F10) was injected into the armpit of the mouse forelimb, 0.1 mL (5×10^6^ cells/mL) of each cell suspension was injected. After the tumor grew to 300 mm^3^ (about 10 days after tumor cell inoculation), mice met the requirements for modeling were randomly divided into compound aluminum sulfate injection group and tumor bearing model control group (Control). In addition, the normal mice that were not inoculated with tumor cells in the same batch were set as the normal unvaccinated group (B). Mice in CAS group were given 0.2 mL compound aluminum sulfate injection by intratumoral injection with a single administration. Mice in group Control were given 0.2 mL 0.9% sodium chloride injection intratumoral injection with a single administration. Group Blank is not processed. Tumor growth and body weight of mice were monitored after administration. The day of tumor cell inoculation was recorded as day 0 (d 0). The tumor volume is calculated as follows: V=L*W^2^/2 (V= tumor volume, L= long diameter, W= short diameter). Tumor growth inhibition rate (tumor weight) = (tumor weight of blank control group - tumor weight of drug administration group)/tumor weight of blank control group *100%, tumor growth inhibition rate (volume) = (volume of blank control group - volume of drug administration group)/volume of blank control group *100%.

To explore the inhibition of blood lung metastasis of melanoma in mice by aluminum sulfate, B16-OVA mouse melanoma cells were used to establish a C57BL/6 mouse subcutaneous transplantation tumor model. 5×10^5^ B16-OVA melanoma cells were inoculated subcutaneously in the armpit of the forelimb of the mice. About 200 mm^3^ after tumor formation, 0.1mL of compound aluminum sulfate injection was injected into the tumor in the experimental group, and 0.1 mL of normal saline was injected into the control group. On the second day after administration, 3×10^5^ (300 microliter μL) B16-OVA mouse melanoma cells were injected into the tail vein to induce lung metastasis, and the growth status of the mice was observed. On the 15^th^ day after the tail vein injection of tumor cells, the mice were performed lung CT using Quantum GX microCT Imaging System (PerkinElmer, Inc, Germany). Then, the mice were euthanized. Lung tissues of the mice were taken, fixed with 4% paraformaldehyde solution, and pathological sections were made for HE staining to observe the metastasis of melanoma in mouse lung.

The RAS-4 Gas Anesthesia System (PerkinElmer, Inc, Germany) was used for animal anesthesia during the procedure, with isoflurane (for pets, Jiangsu Hengfeng Johnson & Johnson, China) as the anesthetic agent.

### 2.3 Fluorescence activated cell sorter analysis

The spleen of mice was extracted from a vertical flow clean bench. The spleen was then cut into small pieces using ophthalmic scissors and placed on a sterilized 200 - mesh stainless steel screen in a plate. Careful grinding was carried out with a 5mL syringe core, and serum - free RPMI1640 culture solution was added during grinding. The liquid under the steel screen was collected, filtered, and placed in a centrifugal tube, after which centrifugation was performed to remove the supernatant. Three milliliters of red blood cell lysate was added and left at room temperature for 5 minutes, followed by centrifugation to remove the supernatant. The cells were suspended with 2mL of culture medium containing 10% fetal bovine serum RPMI 1640. The cells were counted using a cell counter, and 5×10^6^ cells were selected and placed in a flow detection tube. A single cell suspension of tumor tissue was prepared using the same method. An appropriate amount (200 μL) of pre-diluted CD3, CD4, CD8, CD44, NK1.1, F4/80, CD86, CD11b, and Gr - 1 was added into each flow detection tube for flow detection. The antibody information was provided in **Supplementary Table 1**. The same amount of antibody control reagent was added to the blank tube/hole or the same type of tube/hole as the experimental group.

Each tube was incubated in a 4°C refrigerator for 30 minutes in the dark. At least 1 mL of Cell Buffer for staining was added, and then the supernatant was discarded after centrifugation at 350g for 5 minutes. 0.5 mL of fixing solution was added to each tube and incubated at room temperature in the dark for 20 minutes. Centrifugation was carried out to discard the supernatant. The prepared film - breaking liquid was added, followed by centrifugation and discarding of the supernatant, and this process was repeated once. An appropriate amount (200 μL) of pre - diluted CD206 flow detection antibody was added to each flow detection tube and incubated at room temperature for 20 minutes in the dark. 1mL of film - breaking liquid was added, and after centrifugation at 350g for 5 minutes, the supernatant was discarded. The stained cells were re-suspended with 500 microliters of Cell Buffer, screened with a 200 - mesh cell sieve, and then monitored using a flow cytometer (FACSCanto II, BD).

### 2.4 Enzyme linked immunosorbent assay of immune factor secretion

Whole blood of mice was obtained by the method of eyeball extraction. The blood was placed in a centrifuge tube and kept on ice for 2 h. Then it was centrifuged at 2000 rpm for 10 min, and the upper serum was collected for the detection of cytokines for later use.

In a vertical flow clean bench, the spleen of mice was aseptically extracted. The spleen was cut into small pieces with ophthalmic scissors and placed on a sterilized 200 - mesh stainless steel screen, which was then placed in a plate. Careful grinding was carried out with a 5 mL syringe core, and serum - free RPMI1640 culture solution was added during grinding. The liquid under the steel screen was collected, filtered, and placed in a centrifugal tube, after which centrifugation was performed to remove the supernatant. Three milliliters of red blood cell lysate was added and left at room temperature for 5 minutes, followed by centrifugation to remove the supernatant. The cells were suspended with 2 mL of culture medium containing 10% fetal bovine serum RPMI 1640.

Using a cell counter, 5×10^6^ cells were taken and placed in 24 - well plates, and 2 mL of culture medium containing 10% fetal bovine serum RPMI 1640 was added. After being cultured in a cell incubator (37°C, 5% CO_2_) for 72 hours, the culture medium was collected in a centrifuge tube. After centrifugation, the upper culture medium was collected for the detection of cytokines.

The expression of immune factors in the serum and splenic tissue culture upper fluid of mice in each group was detected using the ELISA kits produced by Biolegend, including IL-2 ELISA kit (575409), IL-4 ELISA kit (575609), IL-10 ELISA kit (575809), IL-12 ELISA kit (433607), TNF-α ELISA kit (430901), IFN-γ ELISA kit (430807), and TGF-β ELISA kit (594509).

### 2.5 CD8 and IFN-γ protein expression in tumor tissues

In order to verify the correlation between tumor inhibition mechanism and CD8 and IFN-γ, immunofluorescence (IF) was performed in histopathological sections to detect the expression of CD8 and IFN-γ proteins in different groups of tumor tissues.

### 2.6 Statistical methods

Statistical analysis was performed using GraphPad Prism 10.1 and SPSS 23.0 software. All measurement data were expressed as mean ± standard deviation (SD). For comparisons between two groups, an independent samples t-test was used. For comparisons among multiple groups, one-way analysis of variance (ANOVA) was performed, followed by the least significant difference (LSD) *post hoc* test for pairwise comparisons. A p-value of <0.05 was considered statistically significant.

## 3 Results

### 3.1 The inhibitory effect of compound aluminum sulfate injection on tumor growth

**Figure 1:**
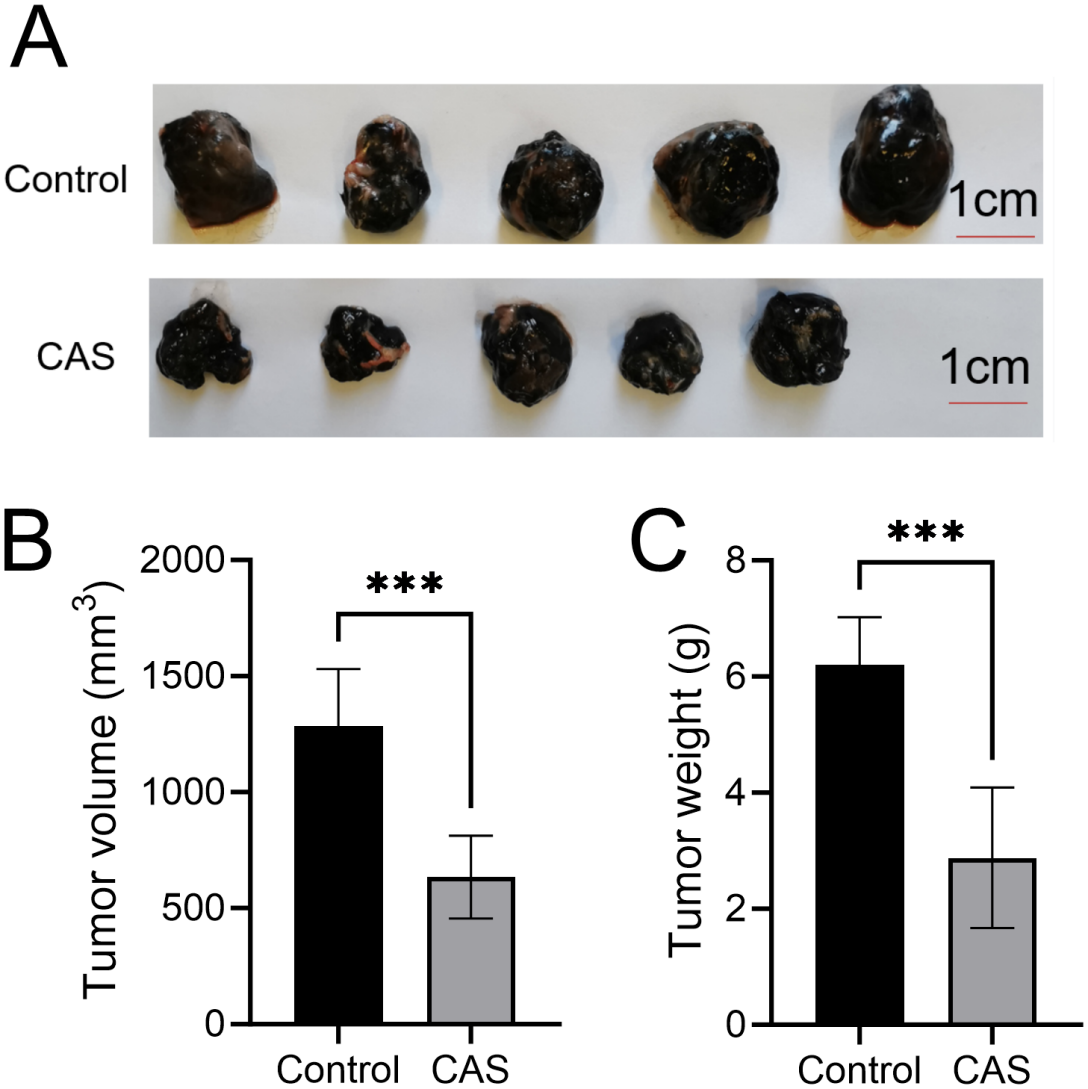
Effect of compound aluminum sulfate injection on tumor volume and tumor weight in transplanted melanoma mice. **(A)** Representative images of tumors excised from control and CAS-treated mice. **(B)** Quantitative analysis of tumor volume in control and CAS groups, measured in cubic millimeters (mm³). **(C)** Quantitative analysis of tumor weight in control and CAS groups, measured in grams (g). Data are presented as mean ± standard deviation (SD). ***P<0.001.

In the syngeneic tumor model, there was no significant difference in body weight among all groups before tumor cell inoculation. Following tumor inoculation, the overall weight of mice in the tumor-bearing groups increased before death. The weight of mice in the tumor-bearing model control group increased significantly compared with normal mice without tumor inoculation and those in the drug administration group (**Figures 1A, B**). In the CAS-treated group, both tumor weight and tumor volume were significantly reduced compared to the tumor-bearing model control group (**Figures 1A–C**). The inhibition rate was 53.5% based on tumor weight and 47.5% based on tumor volume (**Figures 1B, C**). There were no significant differences in net body weight or general health status among all groups, indicating that the compound aluminum sulfate injection is safe for use.

### 3.2 The inhibition of lung metastasis of melanoma in mice by compound aluminum sulfate injection

**Figure 2:**
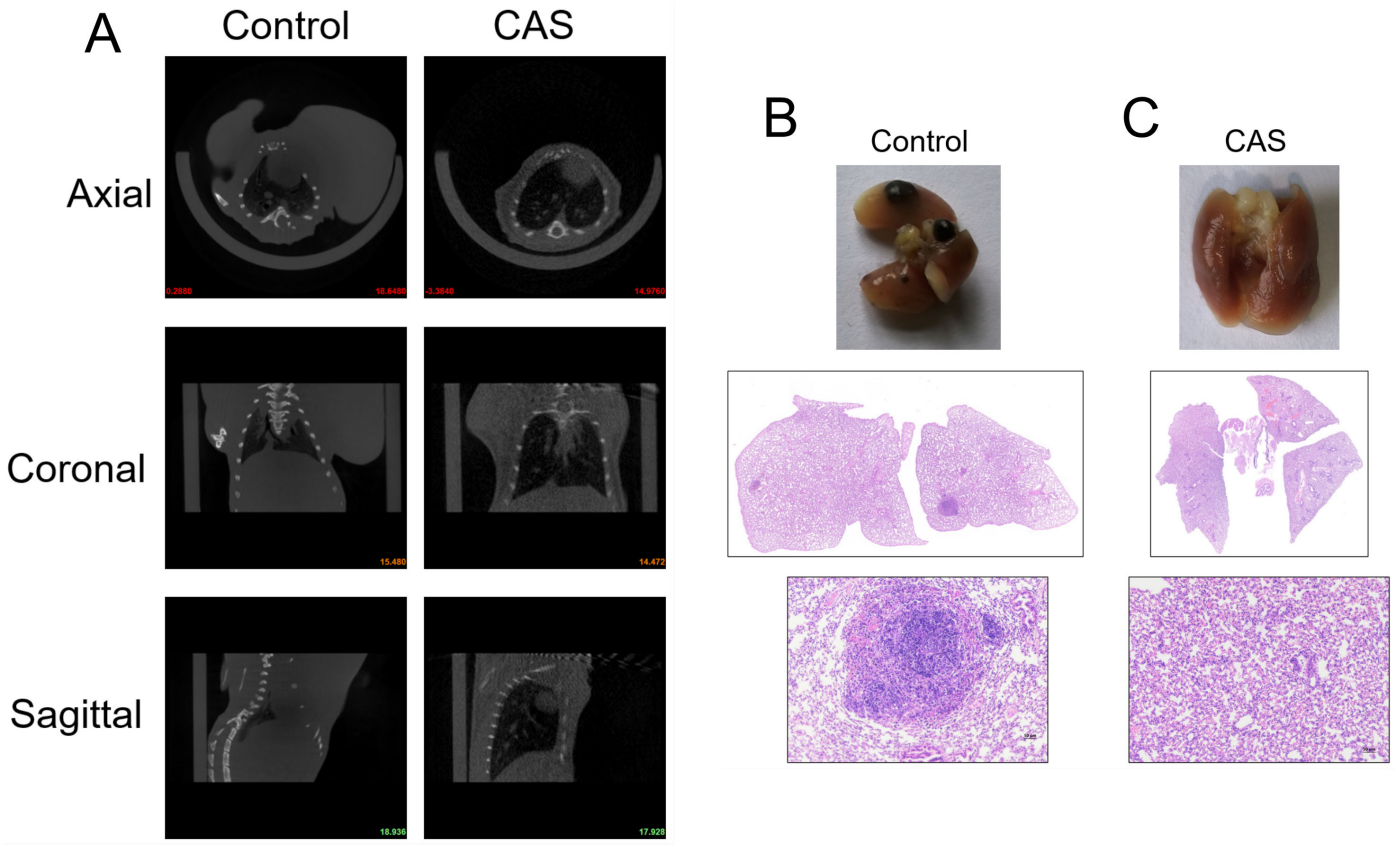
Compound aluminum sulfate injection inhibits hematogenous pulmonary metastasis of melanoma in mice. **(A)** CT images displaying axial, coronal, and sagittal cross-sections of lung tissues from mice in the control group and the CAS group. **(B)** Representative gross morphology of lungs from both the control group and the CAS group. **(C)** Histopathological examination of lung tissue sections stained with hematoxylin and eosin (HE) from the control and CAS-treated groups, showing differences in metastatic foci. Higher magnification images are included to highlight details of the metastatic lesions, n = 6 per group.

In the metastasis model based on B16-OVA mouse melanoma cells, primary tumor growth was significantly inhibited in the group treated with compound aluminum sulfate injection. Lung CT scans and HE staining of lung tissue sections (**Figures 2A–C**) showed a significant reduction in melanoma lung metastasis in the CAS-treated group compared to the control group. These findings suggest that intratumoral injection of compound aluminum sulfate injection can inhibit hematogenous lung metastasis of melanoma in mice.

### 3.3 The effect of compound aluminum sulfate solution on the expression of immune cells in mouse spleen

**Figure 3:**
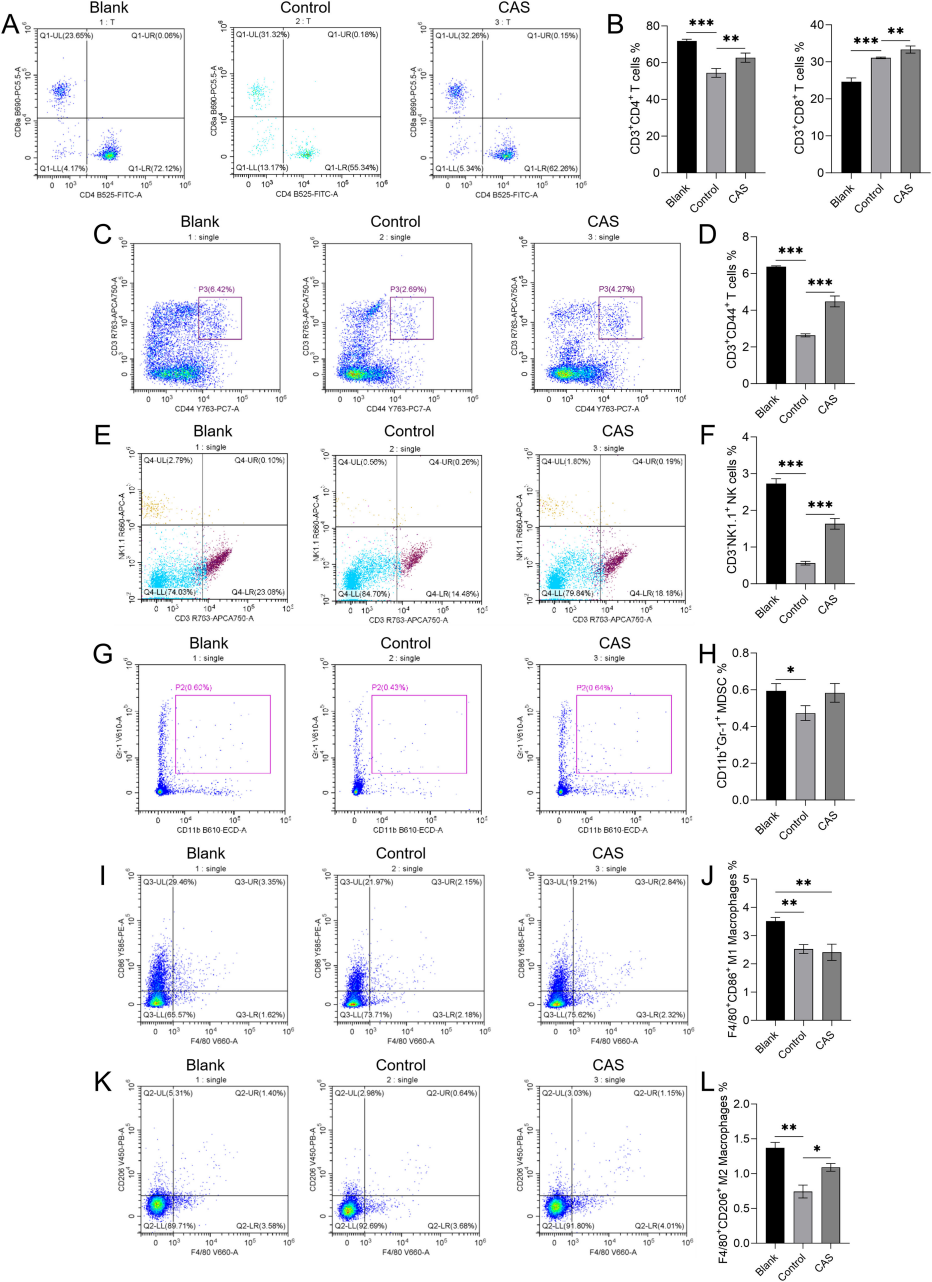
The effect of compound aluminum sulfate (CAS) solution on the expression of immune cells in mouse spleen. The flow cytometry results are shown for the normal group (Blank), the tumor-bearing model control group (Control), and the drug administration group (CAS) from left to right. The flow cytometry plots **(A, C, E, G, I, K)** and corresponding quantification graphs **(B, D, F, H, J, L)** show the percentages of specific immune cell populations in the spleen tissue of different groups. **(A, B)** CD3^+^CD4^+^ Helper T cells and CD3^+^CD8^+^ Cytotoxic T cells; **(C, D)** CD3^+^CD44 ^+^ memory T cells; **(E, F)** Natural killer cells (CD3^-^NK1.1^+^); **(G, H)** myeloid derived suppressor cells (CD11b^+^Gr-1^+^); **(I, J)** M1 macrophages (F4/80^+^CD86^+^); **(K, L)** M2 macrophages (F4/80^+^CD206^+^). N=5 per group. *P<0.05; **P<0.01; ***P<0.001.

The flow cytometry results of spleen tissue were shown in the normal group (Blank), the tumor bearing model control group (Control), and the drug administration group (CAS) from left to right. The results showed that except CD3^+^CD8^+^ T cells, the number of related immune cells in the spleen of transplanted tumor mice was generally reduced compared with that of ungrafted mice, suggesting that tumor cells had a certain inhibitory effect on the immune function of mice after implantation (**Figures 3A–L**). Specifically, local injection of CAS solution significantly increased the populations of CD3^+^CD4^+^ Helper T cells and CD3^+^CD8^+^ Cytotoxic T cells (**Figures 3A, B**), CD3^+^CD44^+^ Memory T cells (**Figures 3C, D**), Natural Killer cells (CD3-NK1.1^+^) (**Figures 3E, F**), and M2 Macrophages (F4/80^+^CD206^+^) (**Figures 3K, L**) when compared to the control group treated with sodium chloride injection. However, no significant changes were observed between the Control and CAS groups for Myeloid-Derived Suppressor Cells (CD11b^+^Gr-1^+^) (**Figures 3G, H**) and M1 Macrophages (F4/80^+^CD86^+^) (**Figures 3I, J**).

### 3.4 Influence of compound aluminum sulfate injection on the expression of immune cells in the transplanted tumor site

**Figure 4:**
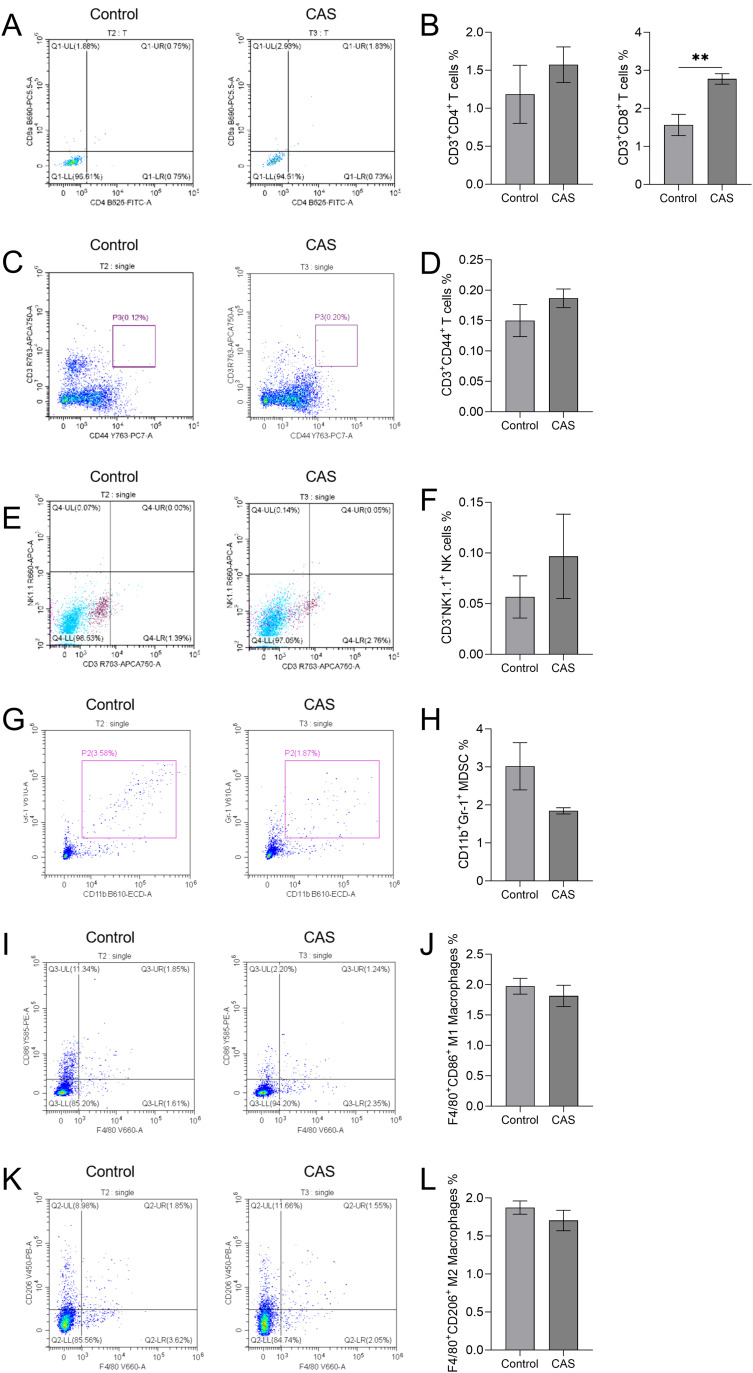
The effect of compound aluminum sulfate (CAS) solution on the expression of immune cells in mouse tumor tissue. The flow cytometry results are shown for the tumor-bearing model control group (Control), and the drug administration group (CAS) from left to right. The flow cytometry plots **(A, C, E, G, I, K)** and corresponding quantification graphs **(B, D, F, H, J, L)** show the percentages of specific immune cell populations in the tumor tissue of different groups. **(A, B)** CD3^+^CD4^+^ Helper T cells and CD3^+^CD8^+^ Cytotoxic T cells; **(C, D)** CD3^+^CD44 ^+^ memory T cells; **(E, F)** Natural killer cells (CD3^-^NK1.1^+^); **(G, H)** myeloid derived suppressor cells (CD11b^+^Gr-1^+^); **(I, J)** M1 macrophages (F4/80^+^CD86^+^); **(K, L)** M2 macrophages (F4/80^+^CD206^+^). N=5 per group. *P<0.05; **P<0.01; ***P<0.001.

Flow cytometry analysis of tumor tissue revealed that local injection of CAS solution significantly increased the infiltration of CD3^+^CD8^+^ T cells within the tumor tissue compared to the control group (P<0.01) (**Figures 4A, B**). No significant changes were observed in the populations of CD3^+^CD4^+^ T lymphocytes (**Figures 4A, B**), memory T cells (CD3^+^CD44^+^) (**Figures 4C, D**), natural killer cells (NK1.1^+^) (**Figures 4E, F**), myeloid-derived suppressor cells (CD11b^+^Gr-1^+^) (**Figures 4G, H**), M1 macrophages (F4/80^+^CD86^+^) (**Figures 4I, J**), or M2 macrophages (F4/80^+^CD206^+^) (**Figures 4K, L**) between the CAS-treated group and the control group (*P*>0.05).

### 3.5 The expression levels of cytokines in splenic tissue culture supernatant and mouse serum

**Figure 5:**
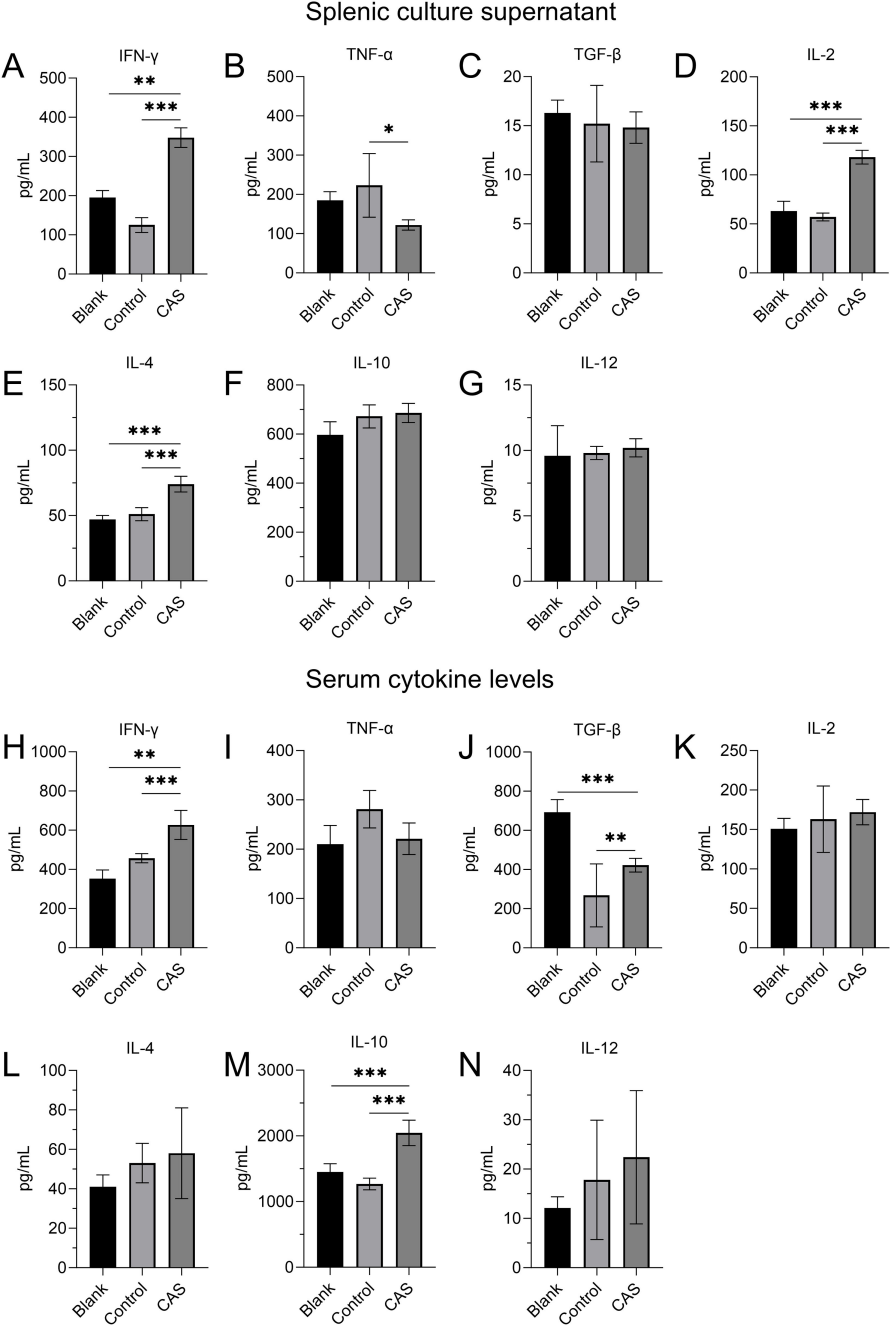
Cytokine expression in splenic culture supernatant and in serum. **(A-G)** Cytokine levels in splenic culture supernatant. **(A)** IFN-γ, **(B)** TNF-α, **(C)** TGF-β, **(D)** IL-2, **(E)** IL-4, **(F)** IL-10, and **(G)** IL-12. **(H-N)** Serum cytokine levels. **(H)** IFN-γ, **(I)** TNF-α, **(J)** TGF-β, **(K)** IL-2, **(L)** IL-4, **(M)** IL-10, and **(N)** IL-12. Data are presented for the blank group (Blank), tumor-bearing model control group (Control), and drug injection group (CAS). *P<0.05, **P<0.01, ***P<0.001.

Cytokines in splenic culture supernatant were detected by ELISA. The results showed that IFN-γ, TNF-α, IL-2, and IL-4 (*P*<0.05) were significantly increased in the CAS group compared to the control group (**Figures 5A, B, D, E**). There were no significant differences in the secretion levels of TGF-β (**Figure 5C**), IL-10 (**Figure 5F**), or IL-12 (**Figure 5G**), between the two groups.

The results of serum cytokine determination showed that compared to the control group, serum levels of IFN-γ (P<0.01), TGF-β and IL-10 were significantly increased in the intratumoral injection of CAS solution group (**Figures 5H, J, M**). The secretion of IL-4 (**Figure 5L**), IL-12 (**Figure 5N**), TNF-α (**Figure 5I**), and TGF-β (**Figure 5J**), and IL-2 (**Figure 5K**) were not significantly affected.

### 3.6 Effects of compound aluminum sulfate injection on the expression of CD8 and IFN-γ proteins in tumor tissues

**Figure 6:**
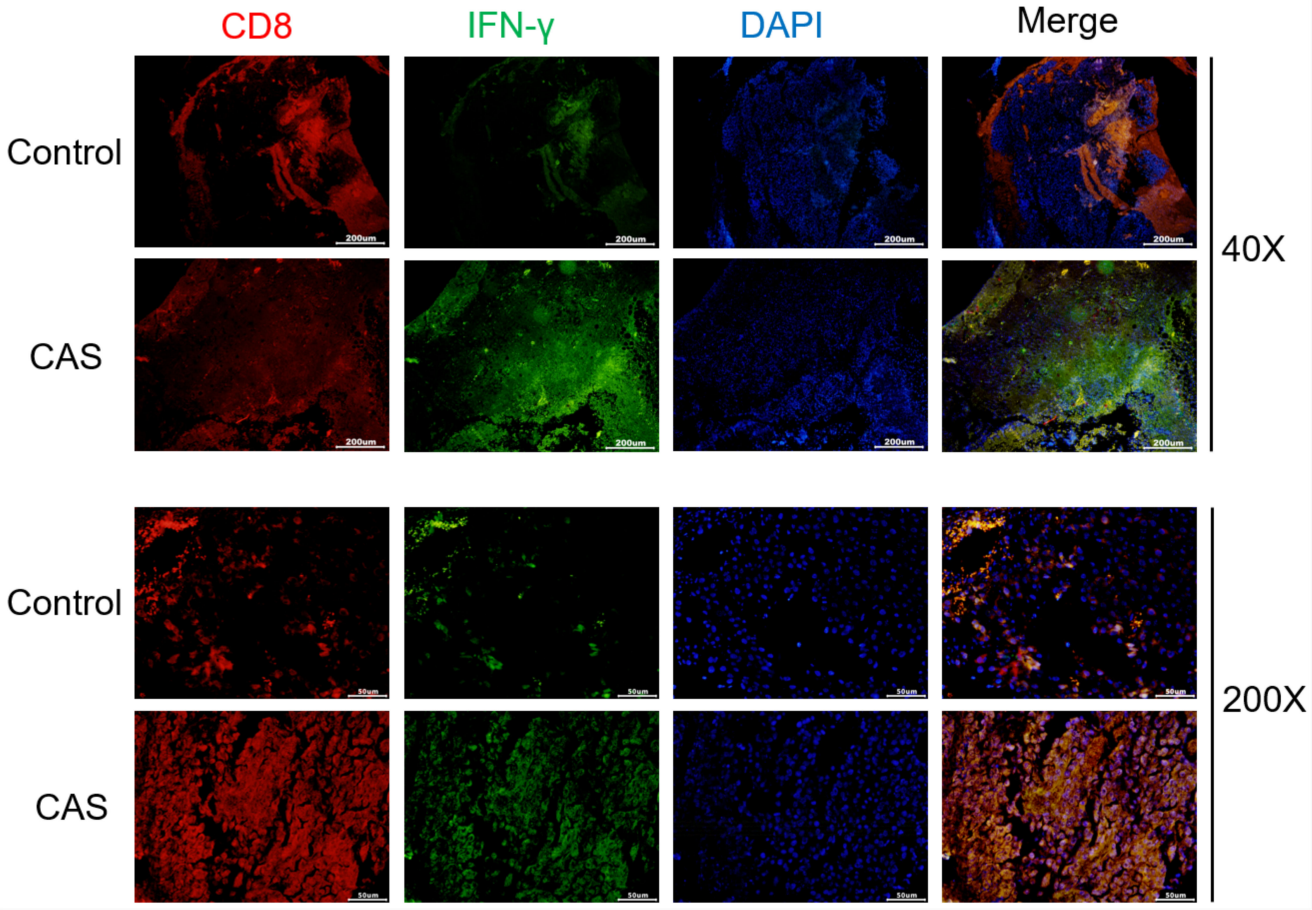
Expression of CD8 and IFN-γ proteins in tumor tissues of control and CAS treatment group (40× and 200×).

The expression of CD8 and IFN-γ proteins in different groups of tumor tissues were detected by immunofluorescence (IF) (**Figure 6**). Compared with the control group, the expression of CD8 and IFN-γ protein in the tumor tissue of the aluminum sulfate solution group was significantly increased.

## 4 Discussion

The present study demonstrates that intratumoral injection of compound aluminum sulfate (CAS) exerts robust anti-tumor effects in murine melanoma models. It significantly inhibiting both primary tumor growth and hematogenous lung metastasis. Importantly, our findings reveal that CAS modulates systemic and local immune responses, enhancing cytotoxic lymphocyte populations and pro-inflammatory cytokine secretion. These results position CAS as a dual-action therapeutic agent, combining direct tumoricidal activity with immunomodulatory properties, and provide mechanistic insights into its potential clinical utility. Consistent with prior reports in bladder cancer models (1), CAS treatment reduced primary tumor volume and weight by 47.5% and 53.5%, respectively. This aligns with the established necrotizing effect of aluminum sulfate, which disrupts cellular integrity through coagulative necrosis (1). However, the significant inhibition of lung metastasis suggests that CAS’s therapeutic benefits extend beyond localized cytotoxicity. Metastatic dissemination is tightly regulated by immune surveillance (21–26). The reduction in pulmonary tumor nodules implies systemic immune activation.

These finding align with studies showing that aluminum oxide nanoparticles (nano-alum) enhance cytotoxic lymphocyte activity and synergize with tumor cell vaccines to suppress metastasis (16, 27–29), suggesting that CAS’s aluminum component may similarly amplify antigen-specific immunity. Flow cytometry revealed that CAS increased splenic CD3^+^CD8^+^ cytotoxic T cells, CD3^+^CD44^+^ memory T cells, and NK cells, indicating systemic immune activation. Notably, CD8^+^ T cells and IFN-γ—a key cytokine for antitumor immunity —were also elevated in tumor tissues, suggesting enhanced cytotoxic lymphocyte infiltration. The aluminum in CAS may similarly act as an adjuvant, promoting APC maturation and Th1-polarized immunity (7, 30–32), as evidenced by elevated serum and splenic IFN-γ, TNF-α, and IL-2. Inflammatory factors are pivotal in disease’s progression (33–36). These cytokines are critical for sustaining cytotoxic T cell and NK cell activity (37, 38), further supporting CAS’s role in bridging innate and adaptive immunity. Recent work on aluminum nanoparticles delivering dual-epitope peptides further underscores the potential of aluminum-based formulations to simultaneously activate CD8^+^ and CD4^+^ T cells (13), which may explain the robust Th1-biased responses observed in our study.

While CAS enhanced immune cell populations systemically, its effects within the TME were more selective. CD8^+^ T cell infiltration increased significantly at the tumor site, but no changes were observed in NK cells, myeloid-derived suppressor cells (MDSCs), or macrophage subsets. This effect may reflect the immunosuppressive nature of the TME, which often resists effector cell infiltration through barriers like PD-L1 upregulation or adenosine accumulation (39–43). Notably, recent advances in aluminum-based nanovaccines demonstrate that co-delivering antigens with dual adjuvants (e.g., CpG-ODN and 3pRNA) can overcome TME immunosuppression by enhancing dendritic cell uptake and cross-presentation (16, 44). The lack of M1 macrophage polarization (F4/80^+^CD86^+^) contrasts with reports using functionalized aluminum adjuvants (45), suggesting that unmodified aluminum compounds like CAS may insufficiently reprogram macrophage phenotypes. Nevertheless, the increased CD8^+^ T cells and IFN-γ in tumors shows CAS’s ability to partially overcome TME immunosuppression, likely through aluminum-driven Th1 activation. Emerging strategies, such as autophagy-activating aluminum hydroxide nanovaccines, further highlight how aluminum formulations can enhance antigen cross-presentation by promoting lysosomal escape and dendritic cell maturation (14, 44), a mechanism that warrants exploration in future CAS studies.

CAS induced a mixed cytokine profile, elevating both pro-inflammatory (IFN-γ, TNF-α, IL-2) and regulatory (IL-4, TGF-β, IL-10) factors. The Th1-associated cytokines IFN-γ and TNF-α are hallmarks of effective antitumor immunity (46–48), enhancing MHC-I expression and T cell cytotoxicity (49). However, the concurrent rise in IL-4 (Th2) and TGF-β suggests a counterregulatory response, potentially limiting excessive inflammation. Similar duality has been observed with aluminum adjuvants, which transiently induce IL-4 to support humoral immunity while maintaining Th1 dominance (13). Notably, TGF-β and IL-10 are typically immunosuppressive, but in this context, they may promote tissue repair post-necrosis or regulate immune homeostasis. This duality mirrors findings from alum-TLR7 agonist conjugates, where balanced Th1/Th2 responses improved anti-MUC1 antibody production and CD8^+^ T cell memory (40, 50), suggesting that CAS’s mixed cytokine profile could be optimized by integrating additional adjuvants.

While this study demonstrates the immune modulation effects of CAS, the precise molecular mechanisms underlying these effects warrant further exploration. Several potential pathways and molecular targets can be considered. For example, the elevated levels of IFN-γ, TNF-α, and IL-2 suggest activation of the JAK-STAT and NF-κB signaling pathways, which are critical for cytotoxic T cell and NK cell activity (51). Additionally, CAS may promote antigen cross-presentation by activating dendritic cells through pathways such as Dectin-1 signaling. Future studies could investigate whether CAS modulates immune checkpoints (e.g., PD-1/PD-L1) or metabolic pathways (e.g., adenosine signaling) that suppress effector cell infiltration. Furthermore, the role of CAS in inducing autophagy-related pathways, such as AMPK-mTOR or Beclin-1 signaling, could provide further mechanistic insights.

This study employed a single-dose CAS injection for experimentation. While this design offers preliminary validation of its anti-tumor and immunomodulatory effects, it falls short of comprehensively evaluating long-term efficacy and the establishment of immune memory. Future research should explore multi-dose regimens and assess their impact on immune memory formation (52). Furthermore, dose-dependency experiments would aid in determining the optimal therapeutic dose and safety profile of CAS. Although this study observed that CAS significantly enhances CD8^+^ T cell infiltration and Th1-type cytokine secretion, its specific molecular mechanisms remain unclear. Future investigations could utilize gene knockout models (e.g., APC-related gene knockout mice) or transcriptomic analysis to further elucidate the adjuvant mechanism of CAS.

## 5 Conclusion

This study positions compound aluminum sulfate injection as a multifaceted anti-tumor agent, exhibiting both direct necrotic and immunomodulatory effects. By increasing CD8^+^ T cell infiltration, enhancing systemic NK cell activity, and promoting Th1-associated cytokines, CAS has the potential to reshape the immune landscape to suppress both primary and metastatic tumors. These findings highlight the therapeutic potential of aluminum-based compounds in oncology, particularly when engineered to optimize immune activation.

## Data Availability

The raw data supporting the conclusions of this article will be made available by the authors, without undue reservation.

## Supplementary material

Supplementary Table 1 Antibodies for flow-cytometry.
