# Supplementary material for: Anti-tumor effect and immune-related mechanism study of compound aluminum sulfate injection in transplanted tumor-bearing mice
Source: Front Immunol. 2025 May 1;16:1583275. doi: 10.3389/fimmu.2025.1583275 (PMC12078244; doi:10.3389/fimmu.2025.1583275)
Supplement: Supplementary Table 1 — Antibodies for flow-cytometry. [file Table1.docx]

**Supplementary Table 1. Antibodies used for flow cytometry analysis**

| **Antibody** | **Manufacturer** | **Cat No.** | **Dilution Information** |
| --- | --- | --- | --- |
| APC/Cyanine7 anti-mouse CD3 | BioLegend | 100220 | 1:200 |
| FITC anti-mouse CD4 | BioLegend | 100406 | 1:200 |
| PerCP/Cyanine5.5 anti-mouse CD8a | BioLegend | 100734 | 1:200 |
| PE/Cy7 anti-mouse/human CD44 | BioLegend | 103029 | 1:200 |
| APC anti-mouse NK-1.1 | BioLegend | 108709 | 1:200 |
| PE anti-mouse CD86 | BioLegend | 105008 | 1:200 |
| Brilliant Violet 421™ anti-mouse CD206 (MMR) | BioLegend | 141717 | 1:200 |
| Brilliant Violet 650™ anti-mouse F4/80 | BioLegend | 123149 | 1:200 |
| Brilliant Violet 605™ anti-mouse Ly-6G/Ly-6C (Gr-1) | BioLegend | 108439 | 1:200 |
| PE/Dazzle™ 594 anti-mouse/human CD11b | BioLegend | 101255 | 1:200 |
| CD8 antibody (Rabbit pAb) | Beijing bioss | bs-0648R | 1:200 |
| IFN-γ antibody (Rabbit pAb) | Beijing bioss | bs-0388R | 1:200 |
